# The Effects of Glucosamine and Chondroitin Sulfate on Gut Microbial Composition: A Systematic Review of Evidence from Animal and Human Studies

**DOI:** 10.3390/nu11020294

**Published:** 2019-01-30

**Authors:** Anna Shmagel, Ryan Demmer, Daniel Knights, Mary Butler, Lisa Langsetmo, Nancy E. Lane, Kristine Ensrud

**Affiliations:** 1 Division of Rheumatic and Autoimmune Diseases, the University of Minnesota, Minneapolis, MN 55455, USA; 2 Division of Epidemiology and Community Health, the University of Minnesota, Minneapolis, MN 55455, USA; demm0009@umn.edu; 3 Department of Computer Science and Engineering and the Biotechnology Institute, the University of Minnesota, Minneapolis, MN 55455, USA; dknights@umn.edu; 4 Division of Health Policy and Management, the University of Minnesota, Minneapolis, MN 55455, USA; butl0092@umn.edu; 5 Division of Epidemiology and Community Health, the University of Minnesota, Minneapolis, MN 55455, USA; langs005@umn.edu; 6 Center for Musculoskeletal Health and Department of Medicine and Rheumatology, the University of California Davis, Davis, CA 95616, USA; nelane@ucdavis.edu; 7 Department of Medicine, the University of Minnesota, Minneapolis, MN 55455, USA; ensru001@umn.edu; 8 Minneapolis VA Center for Chronic Disease Outcomes Research, Minneapolis, MN 55417, USA

**Keywords:** chondroitin, glucosamine, microbiome

## Abstract

Oral glucosamine sulfate (GS) and chondroitin sulfate (CS), while widely marketed as joint-protective supplements, have limited intestinal absorption and are predominantly utilized by gut microbiota. Hence the effects of these supplements on the gut microbiome are of great interest, and may clarify their mode of action, or explain heterogeneity in therapeutic responses. We conducted a systematic review of animal and human studies reporting the effects of GS or CS on gut microbial composition. We searched MEDLINE, EMBASE, and Scopus databases for journal articles in English from database inception until July 2018, using search terms microbiome, microflora, intestinal microbiota/flora, gut microbiota/flora and glucosamine or chondroitin. Eight original articles reported the effects of GS or CS on microbiome composition in adult humans (four articles) or animals (four articles). Studies varied significantly in design, supplementation protocols, and microbiome assessment methods. There was moderate-quality evidence for an association between CS exposure and increased abundance of genus *Bacteroides* in the murine and human gut, and low-quality evidence for an association between CS exposure and an increase in *Desulfovibrio piger* species, an increase in *Bacteroidales S24-7* family, and a decrease in *Lactobacillus*. We discuss the possible metabolic implications of these changes for the host. For GS, evidence of effects on gut microbiome was limited to one low-quality study. This review highlights the importance of considering the potential influence of oral CS supplements on gut microbiota when evaluating their effects and safety for the host.

## 1. Introduction

Glucosamine sulfate (GS) and chondroitin sulfate (CS) are widely marketed as joint-protective supplements, and have been extensively studied for the management of osteoarthritis, with mixed results [1,2,3,4]. GS is a ubiquitous sulfated monosaccharide found in shellfish exoskeletons and in fungi; it can also be produced from plants by fermentation. GS is a key building block for glycosaminoglycans in the extracellular matrix of cartilage and other connective tissues. CS is a complex polysaccharide (glycosaminoglycan) composed of repeating disaccharide chains (glucuronic acid and N-acetylgalactosamine) with sulfate groups in various locations, depending on the source of CS. CS is a structural component of cartilage, providing resistance to compression. CS supplements are produced from bovine, porcine, and marine cartilage.

Oral GS and CS supplements are thought to have anti-inflammatory and antiapoptotic effects on articular cartilage and bone [5,6]. However, only 10–12% of GS and 5–15% of CS are absorbed from the gut [7,8,9]. Absorption of CS from the small intestine is so low that it has been studied as a promising coating agent for drug delivery to the colon [10]. Once chondroitin reaches the cecum, it must be degraded by the gut bacteria to disaccharides in order to be absorbed [11]. GS, as a monosaccharide, does not require bacterial processing for absorption; however, gut bacteria consume more than 50% of orally administered GS before it can be absorbed [7]. Further, the absorbed fraction varies with antibiotic use, suggesting that gut microbiome plays an important role in the bioavailability of GS and CS to the host.

Since GS and CS are used by gut bacteria [12], their therapeutic effects may be exerted through gut bacterial pathways. For example, GS and CS are substrates for sulfate-reducing bacteria, which are implicated in the synthesis of anti-inflammatory compounds and are currently under active investigation for prevention and treatment of several inflammatory and metabolic diseases [13,14,15]. Glucosamine and chondroitin are also important components of intestinal mucin, acting as a barrier between gut flora and the intestinal wall, and potentially affecting gut permeability and intestinal immune mediation [16,17,18]. Understanding the effects of GS and CS on gut microbiota might provide insight into their mechanisms of action and help explain their varied effectiveness in osteoarthritis studies. Hence, we sought to systematically review current evidence of glucosamine and chondroitin sulfate effects on the gut microbiome composition.

## 2. Materials and Methods

Search strategy: This review followed the Preferred Reporting Items for Systematic Reviews and Meta-Analyses (PRISMA) guidelines. We searched Web of Science, MEDLINE, EMBASE, and Scopus databases for articles in English published in peer-reviewed journals and indexed up until July 2018. We used multiple search terms capturing microbiome, microflora, intestinal microbiota/flora, gut microbiota/flora and glucosamine or chondroitin concepts (Appendix A). Search terms were reviewed with an experienced librarian.

Population, interventions, comparisons: We included original studies involving adult humans (age 18 years or older) or other adult mammals, and reported effects of chondroitin sulfate or glucosamine sulfate on the gut microbiome in vivo or in vitro. Any comparator was permitted. We excluded studies that evaluated n-acetyl glucosamine, as it is not typically used as a supplement. We also excluded studies of mixed interventions, such as combined prebiotic and probiotic formulations of CS or GS with other starches, bacteria, or bacterial products.

Outcomes: Key outcomes of interest were differences in the total gut microbial diversity, and absolute or relative abundance of individual microbial species after exposure to CS or GS, when compared with baseline value or control. We formatted results to universal taxonomy from phylum level to lowest available taxonomic level using the NCBI Taxonomy browser (https://www.ncbi.nlm.nih.gov/Taxonomy/Browser/wwwtax.cgi).

Study selection and data extraction: Two authors (AS and RD) screened titles and abstracts for inclusion. Articles selected for full-text review were discussed by all co-authors for final inclusion. Data were extracted into pre-specified structured tables. Missing data points were marked “unknown” or “not reported”.

Summary measures: We performed a qualitative synthesis of findings from included studies. Overall gut bacterial diversity and relative abundance of genera were summarized as “increased,” “decreased,” or “unchanged”. For studies that reported changes in relative abundances of gut bacterial genera, but not statistical significance, we included those genera only if their relative abundance changed at least two-fold. We did not request or analyze data not included in the published reports.

Risk of bias and quality of evidence assessment: The SYRCLE risk of bias tool from the Cochrane collaboration was used for animal studies, and the standard Cochrane tool was used for human studies. The SYRCLE tool evaluates risk of bias using the same criteria as the Cochrane tool for human studies, but adds additional criteria specific to animal studies [19]. Quality of evidence was assessed using CERQual methodology [20]. The CERQual tool has been developed by the Cochrane collaboration for reviews of qualitative evidence and topics with limited knowledge. Assessments were performed by a single author (AS) with team consensus by all authors.

## 3. Results

### 3.1. Study Selection

**Figure 1:**
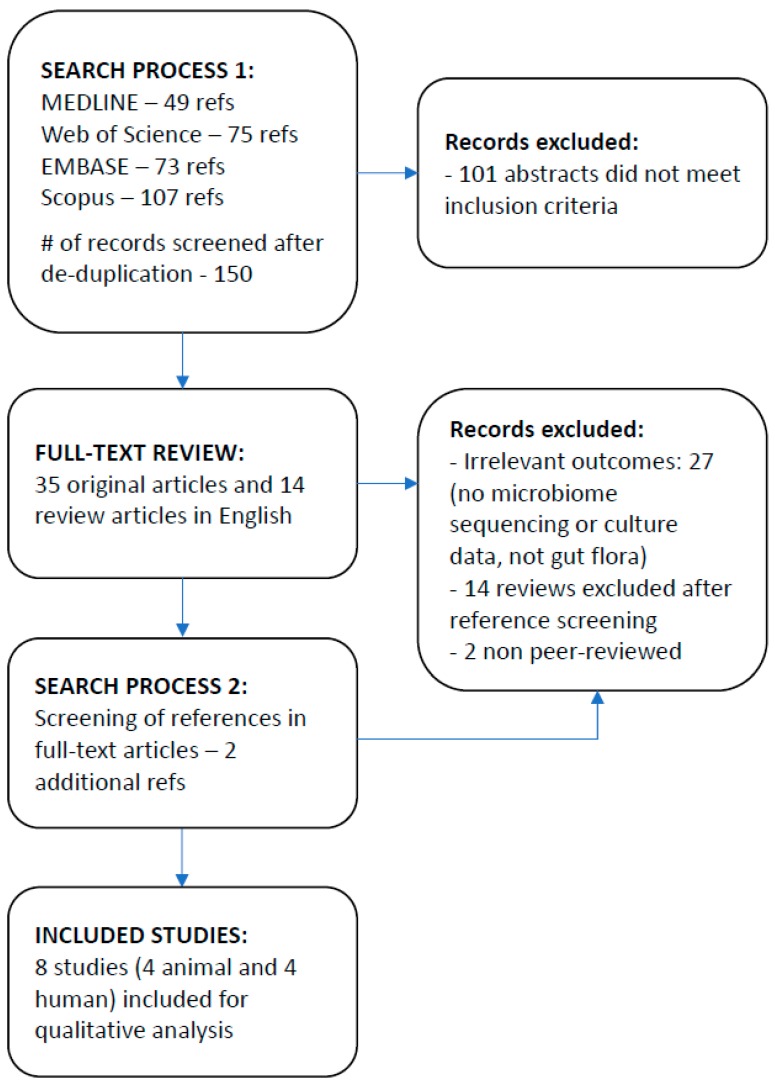
Study flow diagram.

Forty-nine relevant abstracts were identified through MEDLINE search, 73 through EMBASE, 75 through Web of Science, and 107 through Scopus (Figure 1).

### 3.2. Study Characteristics

Of the eight included studies, four were mouse studies [21,22,23,24] and four were human studies [25,26,27,28] (Table 1). In all mouse studies, controlled feeding with CS was carried out, and gut microbial composition assessed after the feeding period. Background diets differed between mouse studies, and included a standard maintenance diet in studies by Liu et al. [21] and Shang et al. [22], and lower fermentable carbohydrate/high fat diets in studies by Pichette et al. [23] and Rey et al. Baseline body weight of the mice was not specifically described in any of the studies. One of the mouse studies used artificial microflora [24]. Human studies were more heterogeneous, with three of four performed in vitro using fecal material from healthy volunteers and CS growth media [25,26,27]. The fourth human study was a clinical trial of GS vs. CS-containing supplement in a knee osteoarthritis population [28]. Sources of CS also varied widely in human studies (fucosylated chondroitin from sea cucumber, purified chondroitin sulfate media, green lipped mussel extract). Given the substantial differences in methods and the reporting of outcomes, pooling of results was not feasible.

### 3.3. Results of Individual Studies

Four studies reported the effect of CS on total gut microbial diversity (Table 2). Two mouse studies and one human study showed no significant change in overall diversity of species after CS supplementation [21,22,28]. One in vitro human study reported a decrease in the Shannon diversity index in two of three donor fecal samples, and no change in the third, when exposed to CS as a single carbon source [27]. There were concordant changes between studies in the abundance of individual gut microbes after CS exposure. The most consistent effect shown in two animal and three human studies was an increase in the relative abundance of genus *Bacteroides* [21,22,26,27,28]. Additionally, two human studies showed a decrease in *Lactobacillus* genus after CS exposure [25,28], two mouse studies showed a relative increase in *Bacteroidales S24-7* family [21,22], and two mouse studies that investigated the bacterium *Desulfovibrio piger* reported an increase in its relative abundance following CS feeding [23,24]. The abundance of *Clostridium* genus increased in two human studies [25,26], however, one human study reported a decrease [28].

Several possible sources of variability in the response to CS supplementation emerged in this literature review. Three of four mouse studies included only male mice [21,23,24], but the one mouse study that used both male and female mice reported marked differences in the baseline gut microbial composition between males and females, as well as sex differences in the response to CS feeding [22]. One small human study also reported variation among six Asian individuals of different ages and sexes in their ability to degrade CS [25]. In this study, fecal samples from three of six individuals did not contain any bacteria able to ferment fucosylated chondroitin sulfate in vitro. Finally, studies used different CS sources and isoforms (bovine vs. marine CS, and different sulfate group positioning on the chondroitin molecule). Two mouse studies that directly compared different CS isoforms found differences in their effects on gut microbiota [21,22].

Only one human study evaluated the effects of GS on the gut microbiota (Coulson et al. [28]). This study found no significant differences in the total gut microbial diversity after GS supplementation, but reported decreased absolute abundance of *Staphylococcus*, *Enterococcus*, and *Clostridium* genera after supplementation (Table 2).

### 3.4. Risk of Bias

**Figure 2:**
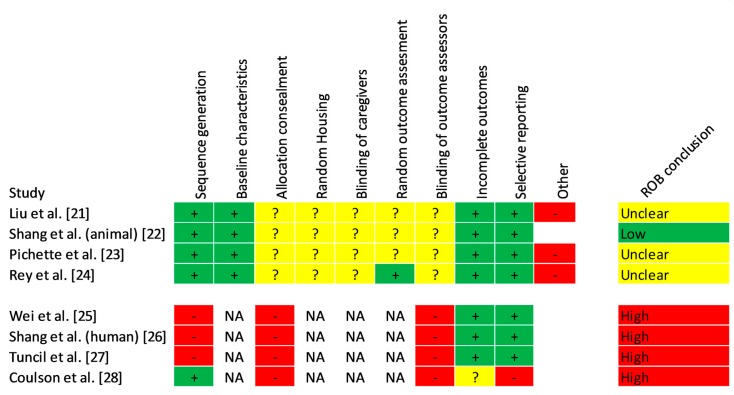
Risk of bias. ROB—risk of bias. NA—not applicable. The risk of bias was rated as “unclear” in three out of four animal studies. While all studies randomized animals into groups, and presented the baseline characteristics and outcomes appropriately, none of the studies reported on allocation concealment, random housing, blinding of the caregivers, or blinding and randomization of outcome assessment. Additionally, three out of four studies were downgraded for using only male animals. In human studies, the risk of bias was high in three non-randomized, non-blinded experimental studies that used convenience sampling and had small sample sizes, as well as in the fourth study, a non-blinded randomized controlled trial.

The risk of bias was rated as “unclear” in three of four animal studies (Figure 2). All studies randomized animals into groups, and presented baseline characteristics and outcomes appropriately. However, none of the animal studies reported on allocation concealment, random housing, blinding of the caregivers, or blinding and randomization of outcome assessment. Additionally, three of four studies were downgraded for using only male animals [21,23,24]. In human studies, the risk of bias was high in three non-randomized, non-blinded experimental studies that used convenience sampling and had small sample sizes [25,26,27], as well as in the fourth study, a non-blinded randomized controlled trial [28].

### 3.5. Synthesis of Results

Based on the overall body of evidence, the confidence in the findings from studies included in this review was moderate to very low (Table 3). There was moderate-quality evidence for an association between CS exposure and increased abundance of genus *Bacteroides* in the murine and human gut, and low-quality evidence for an association between CS exposure and an increase in *Desulfovibrio piger* species, an increase in *Bacteroidales S24-7* family, and a decrease in *Lactobacillus*. Very low-quality evidence suggested that variation in response to CS depended on its source, isoform, and host characteristics. Evidence from one low-quality study was insufficient to draw conclusions for GS effects on gut microbiome.

## 4. Discussion

This systematic review evaluated the evidence for the effects of CS and GS on the gut microbiome. Overall, few high-quality studies were available, and the confidence in the evidence was low for CS and insufficient for GS. However, some concordant results emerged. In several studies, CS supplementation did not alter the overall gut microbial diversity, but affected the abundance of individual bacterial genera. The most consistent finding between heterogeneous animal and human studies was an increase in the abundance of genus *Bacteroides* following exposure to CS in vivo and in vitro.

*Bacteroides* is the most abundant bacterial genus in the human gut, and is enriched in people consuming a “Western” diet high in meat and fat [29,30]. Members of the *Bacteroides* genus are known to digest a wide variety of animal and plant glycans as their primary energy source [31,32]. In the absence of dietary glycans, *Bacteroides* can digest intestinal mucin [33,34], which can lead to inflammation at the intestinal wall, and downstream inflammatory effects on the host [35,36]. Hence, supplemental CS might serve as an exogenous substrate for *Bacteroides*, and protect intestinal mucin from degradation [37]. For example, the Liu et al. [21] study, which demonstrated an increase in *Bacteroides* following CS supplementation, described lower blood lipopolysaccharide (LPS) levels in mice that received CS during exhaustive exercise. This illustrates a possible gut barrier protective effect of CS under exhaustive exercise conditions. Liu et al. [21] further described an increase in fecal short-chain fatty acid production associated with CS supplementation - another possible mechanism of anti-inflammatory and gut-protective effect of CS supplementation in mice. It appears that different species within the *Bacteroides* genus may exhibit different responses to CS [38]. Thus, further studies at species level are needed to fully understand the effects of CS supplementation on gut microflora.

Several members of genus *Bacteroides* secrete sulfatases, and are capable of cleaving sulfate groups from chondroitin sulfate, and other sulfated glycans [39,40]. This not only increases the bioavailability of complex glycans to the host and other gut bacteria, but also releases sulfate for utilization by sulfate reducing bacteria (SRB). Of note, one SRB species, *Desulfovibrio piger*, increased in abundance following CS feeding in two mouse studies. SRB have been of increasing interest in human health, due to their positive effects on weight loss and insulin sensitivity [41], as well as possible detrimental effects in inflammatory bowel disease due to increased H_2_S production [42]. Among studies evaluated in this review, Pichette et al. [23] and Rey et al. both showed that CS supplementation increases the abundance of D. piger and colonic H_2_S levels in mice. Pichette et al. [23] went on to show that these changes are associated with increased GLP1 and insulin secretion, as well as improved oral glucose tolerance. Rey et al. demonstrated that increases in D. piger abundance and colonic H_2_S following CS supplementation did not compromise the gut barrier. These findings suggest beneficial functional effects of D. piger in a setting of CS supplementation.

Other concordant review findings included an increase in the abundance of *Bacteroidales S24-7* family following exposure to CS (two studies), and a decrease in the abundance of *Lactobacillus* (two studies). *Bacteroidales S24-7* is a recently discovered and less characterized family of gut bacteria [43]. Members of this family are known to process plant and host glycans, and are also known to increase in murine models of colitis during treatment-induced remission [44]. The decrease in *Lactobacillus* may be related to anti-adhesion properties of CS, as was suggested in one vaginal microbiome study, where CS isomers A and C markedly reduced adhesion of *Lactobacillus* to epithelial cells [45]. While intriguing, these findings require further validation.

Low to very low-quality evidence showed variation in response to CS depending on its source, isoform, and host characteristics. Different sources of CS are associated with different locations of sulfate groups on the CS molecule; this situation presents a challenge for gut bacteria, as different enzymes are needed to digest different isoforms [46]. Bacteria may adapt and expand their enzyme repertoire, however this process may take time, and may not always make biological sense [47]. For example, in a setting of diverse high-carbohydrate diet, other carbohydrates, like mannose and xylan, may be used preferentially instead of CS [27,48]. Therefore, both host diet and gut microbial composition likely determine the rate of CS degradation and its subsequent therapeutic effects.

It is less clear why CS effects on gut microbiota differed by sex in one mouse study. Previous mouse studies have reported strain-specific sex differences in gut microbial composition among different strains of mice [49]. Sex differences were also observed in human studies, including lower relative abundance of phylum *Bacteroidetes* in women [50]. Interestingly, intra-articular CS concentrations are also reportedly lower in women than in men [51]. The possible sex differences in CS metabolism necessitate further investigation, and inclusion of animals of both sexes in future studies.

Evidence for GS effects on gut microbiome was limited to one low-quality human study. Many similar mechanisms related to degradation of CS apply to GS, including mucin protection [52], sulfate donation, and varied digestion depending on other sources of carbohydrate in the diet and gut microbial composition [53]. However, differences are likely many, since glucosamine is a monosaccharide, and its digestion is likely metabolically “easier” than that of CS. Further studies are needed to understand gut utilization of GS, its local effects, and absorption.

Our review and the included studies had several limitations. Search was limited to peer-reviewed articles in the English language, and therefore may be subject to publication bias. The quality, methodology, and reporting of results were highly variable among included studies, and calculated pooled estimates of results were not obtained. It should also be noted that all mouse studies of CS supplementation used high-dose controlled feeding protocols, which may not be representative of typically lower relative doses of supplemental CS used by humans. All human studies that reported the effects of CS and GS on the gut microbiome were of low quality, which underscores the lack of direct evidence to address this research question in humans.

## 5. Conclusions

This review highlights the importance of considering oral CS interactions with gut microbiota when evaluating its usefulness, bioavailability, and potential adverse effects. The most convincing available evidence reported in several studies is that CS supplementation increases the relative abundance of the gut bacterial genus Bacteroides, which may play important roles in regulating the symbiosis in the gut microbial community, as well as host health. The very limited evidence regarding the effects of chondroitin and glucosamine sulfate on the gut microflora calls for further studies.

## Figures and Tables

**Table 1:** Description of included studies.

		Title	Compound Studied	Dose	Comparator	Duration of Exposure	Participants (*N*)	Microbiome Assessment Method	Microorganism Identification Reference
**Animal Studies**									
1	Liu, F. et al., 2017 [21]	Chondroitin sulfate disaccharides modified the structure and function of the murine gut microbiome under healthy and stressed conditions	CS disaccharides CS-4s and CS-6s	150 mg/kg	PBS + Both groups fed ad libitum Maintenance Purified Diet	16 days	Balb/c male mouse (*N* = 30, randomly assigned to 4 groups)	16S sequencing	QIIME pipeline, GreenGene database
2	Shang, Q et al., 2016 [22]	Structural modulation of gut microbiota by chondroitin sulfate and its oligosaccharide	CS isomers CSA, CSC, CSO	150 mg/kg	Normal saline + Both groups fed standard lab diet	6 weeks	Kunming male and female mouse (*N* = 48, randomly assigned to 8 groups, 6 mice each)	16S sequencing	UPARSE pipeline, database not reported
3	Pichette, J. et al., 2017 [23]	Hydrogen sulfide and sulfate prebiotic stimulates the secretion of GLP-1 and improves glycemia in male mice	CS	3% wt/wt	No supplement + Both groups fed diet low in fermentable carbohydrate	4 weeks	Male wild-type C57BL/6 mouse (*N* = 26, randomly assigned to 2 groups)	Targeted PCR	NA
4	Rey, F. et al., 2013 [24]	Metabolic niche of a prominent sulfate-reducing human gut bacterium	CS	3% wt/wt	No supplement + Both groups fed High fat/high sugar diet	1 week	NMRI gnobiotic male germ-free mouse, artificial humanized microflora (*N* = 20 per group)	COPRO-seq	NA
**Human Studies**									
5	Wei, C. et al., 2017 [25]	In vitro fermentation behaviors of fucosylated chondroitin sulfate from Pearsonothuria graeffei by human gut microflora	Fucosylated CS from sea cucumber	growth media	none	72 h	*n* = 6 healthy human fecal samples/in vitro	16S-based Real-time quantitative PCR	BLAST
6	Shang, Q. et al., 2016 [26]	Degradation of chondroitin sulfate by the gut microbiota of Chinese individuals	CS as a sole carbon source in growth medium	growth media	none	72 h	*n* = 6 healthy human fecal samples/in vitro	16S sequencing	BLAST
7	Tuncil, Y. et al., 2017 [27]	Delayed utilization of some fast-fermenting soluble dietary fibers by human gut microbiota when presented in a mixture	CS as a sole carbon source in growth medium	growth media	none	12 h	*n* = 3 healthy human fecal samples/in vitro	16S sequencing	QIIME pipeline, GreenGene database
8	Coulson, S. et al., 2013 [28]	Green-lipped mussel extract (Perna canaliculus) and glucosamine sulfate in patients with knee osteoarthritis: Therapeutic efficacy and effects on gastrointestinal microbiota profiles	12% CS from green-lipped mussel extract or GS	350 mg of CS/day	3000 mg of GS/day	12 weeks	*n* = 11 men and 29 women (38 total, randomized to green-lipped mussel extract or GS)	MALDI-TOF Mass spectrometry	MALDI Byotyper

CS—Chondroitin Sulfate, GS—glucosamine sulfate, PBS—phosphate-buffered saline, CSA—Chondroitin Sulfate A, CSC—Chondroitin Sulfate C, CSO—Chondroitin Sulfate O.

**Table 2:** Key results: The associations of chondroitin and glucosamine sulfate exposure with gut microbial diversity and abundance of specific microorganisms.

		Total Gut Microbial Diversity	Microorganism						Abundance Change Attributed to Intervention (If Reported) and Direction of Change		
			Phylum	Class	Order	Family	Genus	Species			
**Animal Studies**											
1	Liu et al., 2017 [21]	No change in total number of OTUs, Chao1, Shannon, inverse Simpson indices	*Bacteroidetes*	*Bacteroidia*	*Bacteroidales*	*Bacteroidaceae*	*Bacteroides*	*acidifaciens*	0.12%	0.22%	Increased *
			*Bacteroidetes*	*Bacteroidia*	*Bacteroidales*	*Bacteroidaceae*	*Bacteroides*		0.0007%	0.20%	Increased *
			*Bacteroidetes*	*Bacteroidia*	*Bacteroidales*	*Bacteroidaceae*	*Bacteroides*		0.0004%	0.06%	Increased *
			*Firmicutes*	*Bacilli*	*Bacillales*	*Bacillaceae*	*Lysinibacillus*	*boronitolerans*	0.0000%	0.0031%	Increased *
			*Proteobacteria*	*Alphaproteobacteria*	*Rhizobiales*	*Brucellaceae*	*Pseudochrobactrum*		0.0014%	0.0041%	Increased *
			*Firmicutes*	*Clostridia*	*Clostridiales*				0.01%	0.15%	Increased *
			*Bacteroidetes*	*Bacteroidia*	*Bacteroidales*	*S24-7*			0.02%	0.13%	Increased *
2	Shang et al., 2016 (animal) ^a^ [22]	No consistent difference in the number of OTUs, Chao1, Shannon, Simpson indices	*Bacteroidetes*	*Bacteroidia*	*Bacteroidales*	*Rikenellaceae*	*Alistipes*		increased		
			*Bacteroidetes*	*Bacteroidia*	*Bacteroidales*	*S24-7*			increased in M, decreased in F		
			*Bacteroidetes*	*Bacteroidia*	*Bacteroidales*	*Bacteroidaceae*	*Bacteroides*		decreased in M, increased in F		
			*Proteobacteria*	*Epsilonproteobacteria*	*Campylobacterales*	*Helicobacteraceae*	*Helicobacter*		decreased in M, no change in F		
			*Firmicutes*	*Clostridia*	*Clostridiales*	*Lachnospiraceae*	*NK4A136*		decreased		
3	Pichette et al., 2017 ^b^ [23]	Not studied	*Proteobacteria*	*Deltaproteobacteria*	*Desulfovibrionales*	*Desulfovibrionaceae*	*Desulfovibrio*	*piger*	0.10%	0.13%	Increased *
4	Rey et al., 2013 ^b^ [24]	Not studied	*Proteobacteria*	*Deltaproteobacteria*	*Desulfovibrionales*	*Desulfovibrionaceae*	*Desulfovibrio*	*piger*	2.30%	3.50%	Increased *
**Human Studies**											
5	Wei et al., 2017 [25]	Not studied	*Firmicutes*	*Clostridia*	*Clostridiales*	*Clostridiaceae*	*Clostridium*		increased		
			*Actinobacteria*	*Actinobacteria*	*Bifidobacteriales*	*Bifidobacteriaceae*	*Bifidobacterium*		increased		
			*Bacteroidetes*	*Bacteroidia*	*Bacteroidales*	*Prevotellaceae*	*Prevotella*		increased		
			*Firmicutes*	*Bacilli*	*Lactobacillales*	*Lactobacillaceae*	*Lactobacillus*		decreased		
			*Proteobacteria*	*Gammaproteobacteria*	*Enterobacterales*	*Enterobacteriaceae*			decreased		
6	Shang et al., 2016 (human) ^c^ [26]	Not studied	*Bacteroidetes*	*Bacteroidia*	*Bacteroidales*	*Bacteroidaceae*	*Bacteroides*	*thetaiotaomicron*	increased		
			*Bacteroidetes*	*Bacteroidia*	*Bacteroidales*	*Bacteroidaceae*	*Bacteroides*	*thetaiotaomicron 82*	increased		
			*Bacteroidetes*	*Bacteroidia*	*Bacteroidales*	*Bacteroidaceae*	*Bacteroides*	*ovatus*	increased		
			*Firmicutes*	*Clostridia*	*Clostridiales*	*Clostridiaceae*	*Clostridium*	*hathewayi*	increased		
7	Tuncil, Y. et al., 2017 [27]	Decreased Shannon index in two of three donor samples; no change in 3rd	*Bacteroidetes*	*Bacteroidia*	*Bacteroidales*	*Bacteroidaceae*	*Bacteroides*		increased		
			*Bacteroidetes*	*Bacteroidia*	*Bacteroidales*	*Tannerellaceae*	*Parabacteroides*		Increased 3 to 20-fold *		
8	Coulson et al., 2013 ^d^ [28]	No difference in number of species before and after treatment in both groups	**GLM (Chondroitin Sulfate source) group**								
			*Proteobacteria*	*Gammaproteobacteria*	*Enterobacterales*	*Enterobacteriaceae*			1.93 × 10 ^7^	6.70 × 10 ^7^	increased
			*Firmicutes*	*Clostridia*	*Clostridiales*	*Eubacteriaceae*	*Eubacteria*		8.14 × 10 ^9^	16.10 × 10 ^9^	increased
			*Firmicutes*	*Bacilli*	*Lactobacillales*	*Streptococcaceae*	*Streptococcus*		1.09 × 10 ^7^	3.65 × 10 ^7^	increased
			*Bacteroidetes*	*Bacteroidia*	*Bacteroidales*	*Bacteroidaceae*	*Bacteroides*		1.26 × 10 ^10^	2.05 × 10 ^10^	increased
			*Firmicutes*	*Clostridia*	*Clostridiales*	*Clostridiaceae*	*Clostridium*		2.04 × 10 ^9^	0.95 × 10 ^9^	decreased
			*Firmicutes*	*Bacilli*	*Bacillales*	*Staphylococcaceae*	*Staphylococcus*		4.51 × 10 ^6^	0.02 × 10 ^6^	decreased
			*Firmicutes*	*Bacilli*	*Lactobacillales*	*Enterococcaceae*	*Enterococcus*		1.80 × 10 ^7^	0.66 × 10 ^7^	decreased
			*Yeast*						8.52 × 10 ^3^	4.19 × 10 ^3^	decreased
			*Firmicutes*	*Bacilli*	*Lactobacillales*	*Lactobacillaceae*	*Lactobacillus*		1.08 × 10 ^9^	0.45 × 10 ^9^	decreased
			**Glucosamine Sulfate group**								
			*Firmicutes*	*Bacilli*	*Bacillales*	*Staphylococcaceae*	*Staphylococcus*		7.03 × 10 ^5^	0.48 × 10 ^5^	decreased
			*Firmicutes*	*Bacilli*	*Lactobacillales*	*Enterococcaceae*	*Enterococcus*		6.18 × 10 ^6^	0.63 × 10 ^6^	decreased
			*Firmicutes*	*Clostridia*	*Clostridiales*	*Clostridiaceae*	*Clostridium*		8.22 × 10 ^9^	6.13 × 10 ^9^	decreased

Concordant findings between two or more studies are highlighted with the same color. * Statistically significant finding. For all other findings statistical significance was not reported. a. Results were extracted from a color gradient figure; the top five genera with most visually notable changes were selected. b. *Desulfovibrio piger* was the only bacterium measured in these studies. c. Study selected for CSA-degrading bacteria by culturing stool samples on CSA media. d. Results are reported for a subgroup analysis excluding subjects who took antibiotics or probiotics during the study period. Genera with at least a two-fold change in the mean viable counts were selected.

**Table 3:** CERQual Assessment of Confidence in the Evidence.

Summary of Review Finding	Studies	Methodological Limitations	Coherence	Adequacy	Relevance	*CERQual Assessment*	Explanation of CERQual Assessment
**1. Chondroitin sulfate supplementation has no effect on the overall gut bacterial diversity in mice and humans**	1, 2, 7, 8	Moderate concerns: Studies varied in sampling technique, bacteria identification methods, and in reporting of results.	Moderate concerns: three studies reported concordant results, and one reported a decrease in overall diversity in 2 out of 3 subjects	Moderate concerns: evidence comes from two good quality mouse studies, and two low-quality human studies.	No concerns: The presence of this finding in both mouse and human studies conveys higher relevance.	*Low*	There was moderate coherence among studies, however confidence was downgraded due to moderate concerns about methodology, coherence, and adequacy.
**2. Exposure to Chondroitin sulfate increases the relative abundance of genus *Bacteroides* in mice and humans**	1, 2, 5, 6, 7, 8	Serious concerns: Studies varied widely in sampling technique, chondroitin exposure methodology (in vivo vs in vitro) and in reporting of results.	No concerns: Both mouse studies and three of the four human studies showed coherence in this finding. Fourth human study did not report	Moderate concerns: There were two good quality mouse studies, and four low-quality human studies.	No concerns: The presence of this finding in both mouse and human studies, and in both sexes conveys higher relevance than if it was observed in one species/one sex only.	*Moderate*	There was high coherence among studies, and high relevance, however confidence was downgraded due to serious concerns about methodology and adequacy.
**3. Chondroitin sulfate supplementation may increase the relative abundance of *Desulfovibrio piger in mice***	1, 2, 3, 4	Moderate concerns: All studies used adequate sampling and in-vivo methodology, but one used artificial gut flora.	Serious concerns: Two studies showed an increase in abundance of *D. piger*, one showed no increase, and one showed a decrease.	Serious concerns: The two studies that showed an increase in the abundance of *D. piger* were specifically designed to evaluate *D. piger*.	Moderate concerns: Evidence for this finding comes from mouse studies only, hence relevance to humans is unclear	*Low*	There were minor concerns about methods, coherence between studies was low, and relevance unclear.
**4. Gut microbial response to chondroitin sulfate exposure may vary depending on chondroitin sulfate source and isoform in mice and humans**	1, 2, 3, 5, 6, 8	Serious concerns: The wide range of reported changes in the gut microbiome between studies can be explained by several significant limitations in sampling and microbial identification techniques.	No concerns: All studies reported different groups of bacteria in response to CS exposure	Serious concerns: 1–2 studies per isoform or source of CS	Moderate concerns: finding was observed in both animal and human studies, but human studies had very small sample sizes	*Low*	Given multiple serious limitations in methodology and very low adequacy
**5. Gut microbial response to chondroitin sulfate exposure may vary among sexes and individual subjects in mice and humans**	2, 5	Serious concerns: one of the two studies used a convenience sample of six subjects.	Serious concerns: methods and results were very heterogeneous between the two contributing studies.	Serious concerns: Only one animal and one small in-vitro human study	Moderate concerns: Unclear whether sex differences in mouse microbiome are directly relevant to humans; unclear whether the Chinese human study is relevant to the general population.	*Very low*	Only two studies, possible methodologic explanations for heterogeneity

## Appendix A. Literature Search Algorithms

**Table A1:** Web of Science.

**#5**	#2 AND #1 Refined by: DOCUMENT TYPES: (ARTICLE) AND LANGUAGES: (ENGLISH) *DocType=All document types; Language=All languages;*
**#4**	#2 AND #1 Refined by: DOCUMENT TYPES: (ARTICLE) *DocType=All document types; Language=All languages;*
**#3**	#2 AND #1 *DocType=All document types; Language=All languages;*
**#2**	TOPIC: (glucosamine or chondroitin) *DocType=All document types; Language=All languages;*
**#1**	TOPIC: (microbiom* or microbiot* or microflor* or gut flor* or intestinal flor*) *DocType=All document types; Language=All languages;*

**Table A2:** MEDLINE and EMBASE (via OVID).

#1. (microbiom* or microbiot* or microflor* or gut flor* or intestinal flor*).mp. (mp=title, abstract, heading word, drug trade name, original title, device manufacturer, drug manufacturer, device trade name, keyword, floating subheading word, candidate term word)
#2. (glucosamine or chondroitin).mp. (mp=title, abstract, heading word, drug trade name, original title, device manufacturer, drug manufacturer, device trade name, keyword, floating subheading word, candidate term word)
#3. 1 and 2
#4. limit 3 to English language
#5. limit 4 to article

**Table A3:** Scopus.

(TITLE-ABS-KEY (*microbiom** OR *microbiot** OR *microflor** OR (*gut* AND *flor**) OR (*intestinal* AND *flor**))) AND (TITLE-ABS-KEY (*glucosamine* OR *chondroitin*)) AND (LIMIT-TO (DOCTYPE, *“ar”*)) AND (LIMIT-TO (LANGUAGE, *“English”*))
